# Clinical and Renal Histology Findings and Different Responses to Induction Treatment Affecting the Long-Term Renal Outcomes of Children With ANCA-Associated Vasculitis: a Single-Center Cohort Analysis

**DOI:** 10.3389/fimmu.2022.857813

**Published:** 2022-04-14

**Authors:** Jing Yang, Yuan Yang, Yongli Xu, Lanqi Zhou, Luowen Zhou, Xiaoling Yin, Jinyun Pu, Fengjie Yang, Yaping Liu, Yonghua He, Yaxian Chen, Huiqing Yuan, Liru Qiu, Yu Zhang, Yu Chen, Tonglin Liu, Jinhui Tang, Jianhua Zhou

**Affiliations:** ^1^Department of Pediatrics, Tongji Hospital, Tongji Medical College, Huazhong University of Science and Technology, Wuhan, China; ^2^Department of Neurosurgery, Tongji Hospital, Tongji Medical College, Huazhong University of Science and Technology, Wuhan, China

**Keywords:** ANCA-associated vasculitis, children, remission-induction treatment, ESRD, progression, renal survival

## Abstract

**Introduction:**

Antineutrophil cytoplasmic antibody (ANCA)-associated vasculitis (AAV) is relatively rare in children. This article aimed to analyze clinical and renal histology findings and different responses to induction treatment associated with the long-term renal outcomes in children with AAV in a single center.

**Methods:**

All pediatric patients with AAV admitted to Tongji Hospital from January 2002 to January 2021 were included in the study. The demographic, clinical, pathological, laboratory, and treatment data and outcomes were collected and analyzed to identify predictors associated with response to induction treatment and progression to end-stage renal disease (ESRD).

**Results:**

In total, 48 children with AAV were included in this cohort; 81.25% of them were women, and 91.7% were microscopic polyangiitis (MPA). Kidney involvement was found in 45 patients (93.75%). The most common histopathological subtype was crescentic form in this cohort according to Berden’s classification. In total, 34 patients (70.8%) showed eGFR <60 ml/min/1.73 m^2^ at the time of diagnosis. Complete and partial remission was achieved in 8 patients (16.7%) and 19 patients (39.6%), respectively, following 6-month induction treatment. Half of the patients eventually progressed to ESRD at a mean time of (13.04 ± 15.83) months after diagnosis. The independent predictors of nonremission following induction treatment and progression to ESRD were baseline eGFR <60 ml/min/1.73 m^2^ and hypertension at diagnosis. Renal survival significantly decreased over time in patients with renal sclerotic subtypes or those with nonremission following induction treatment by Kaplan–Meier curve estimation.

**Conclusions:**

Our study demonstrates that women, MPA, and crescentic subtypes are predominant in pediatric AAV in China. Initial renal failure (eGFR <60 ml/min/1.73 m^2^), hypertension, sclerotic pathological subtype, and nonremission following induction treatment are predictive of long-term renal outcomes.

## Introduction

Antineutrophil cytoplasmic antibody (ANCA)-associated vasculitis (AAV) is a multisystemic autoimmune disease characterized by a necrotizing inflammation in the small to medium vessels. According to the 2012 Chapel Hill International Consensus Conference nomenclature of vasculitis (CHCC) (1), AAV includes microscopic polyangiitis (MPA), granulomatosis with polyangiitis (GPA, formerly known as Wegener’s granulomatosis), and eosinophilic granulomatosis with polyangiitis (EGPA, also known as Churg–Strauss syndrome). MPA is a kind of necrotizing vasculitis predominantly affecting the kidney. GPA is a necrotizing granulomatous vasculitis with more sinus and pulmonary involvement, but necrotizing glomerulonephritis is also common. EGPA is an eosinophil-rich and necrotizing granulomatous vasculitis affecting the respiratory tract. In addition, nervous system, skin, mucous, ocular, and cardiovascular involvement can occur in all kinds of AAV.

AAV is associated with the occurrence of circulating autoantibodies against myeloperoxidase (MPO), proteinase 3 (PR3), and other uncommon antigens. MPO-ANCA is common in patients with MPA and usually occurs as perinuclear immunofluorescence patterns (P-ANCA), while PR3-ANCA is commonly associated with GPA and presents as cytoplasmic immunofluorescence patterns (C-ANCA) (2). AAV mainly occurs in adults with an annual incidence of 13–20 cases/million in Europe. However, they are very rare in children with an estimated incidence of 1–6 cases per million (3). A combination of immunosuppression and glucocorticoids remains the mainstream treatment and has saved the lives of a lot of patients with AAV. Despite strong immunotherapy, many AAV patients still died of ESRD, infectious or cardiovascular complications, etc. According to previous reports (4–10), 20% to 45% of children with AAV eventually progressed to ESRD. A recent multicenter study of 85 children with AAV in Italy and Canada (9) has not found any independent prognostic factors of renal outcome in multivariable Cox regression analysis. In a French national study of 66 children with AAV (11), low baseline eGFR levels, non-Caucasian ethnicity, and sclerotic or mixed histopathological patterns were associated with the occurrence of ESRD. Since AAV in children is rare, risk factors of renal long-term outcomes have not been extensively studied. So far, there have been a few reports on the risk factors for renal prognosis in Chinese children with AAV, and there has been no study on the effect of induction therapy on renal long-term outcomes. Hence, the present study is intended to provide such information through analyzing the demographic, clinical, pathological, laboratory, and follow-up data in children with AAV in a single pediatric center.

## Materials and Methods

### Patients

This cohort included all AAV patients hospitalized from January 2002 to January 2021 at the Pediatric Department of Tongji Hospital and younger than 18 years old at diagnosis. The diagnosis of AAV was established according to the Pediatric Rheumatology European Society (PRES) criteria for childhood vasculitis and the 2012 revised Chapel Hill consensus conference nomenclature of vasculitis. Some patients were included in the study due to typical pauci-immune necrotizing glomerulonephritis in renal pathology, although the ANCA tested negative (IF or ELISA for MPO, PR3). AAV secondary to drugs and other autoimmune diseases such as systemic lupus erythematosus (SLE), IgA-associated vasculitis, and rheumatoid arthritis, and primary kidney diseases such as membranous nephropathy and IgA nephropathy were all excluded in this study.

The study was approved by the Ethics Committee of Tongji Hospital and conducted in accordance with the Declaration of Helsinki.

### Data Collection, Treatment, and Definition in Evaluation

The demographic, clinical, pathological, and laboratory treatment data and renal outcomes were collected for analysis. Baseline data included sex, age, height, time from onset to diagnosis, baseline serum creatinine, 24 h urinary protein amount, ESR, PVAS score, ANCA types (immunofluorescence detection), MPO and PR3 titers, remission-induction treatment approaches, and stages of CKD.

The remission-induction treatment was conducted with glucocorticoids and monthly intravenous cyclophosphamides (CYC) at a dosage of 0.5–0.75g/m^2^ for up to 6 months (12). For those presented with eGFR <60 ml/min/1.73 m^2^, plasma exchange (three to five times within 2 weeks) and/or rituximab (at a dosage of 375 mg/m^2^ for 2–4 times) were considered in addition to glucocorticoids and monthly intravenous cyclophosphamides (CYC). Normally, response to induction treatment was assessed after 6 months. After remission-induction therapy, patients were on maintenance treatment with glucocorticoids and mycophenolate mofetil (MMF) or AZA in most cases, only a few patients were on intravenous CYC every 3 months. All patients were evaluated monthly during remission-induction treatment and thereafter every 2 to 4 months during maintenance treatment.

Data were collected and analyzed at the time of diagnosis, after 6-month remission-induction treatment, and at the observation endpoint or the final follow-up.

### Definition

Renal symptoms or parameters at presentation were defined as follows: nephrotic-ranged proteinuria was defined as the 24-h urine protein excretion of more than 40 mg/h/m^2^ or urine protein/Cr ≥2.0 (mg/mg). eGFR was calculated by using the modified Schwartz formula; CKD was defined and classified according to the KDOQI standards; the observation endpoint was defined as arrival of CKD stage V or the last follow-up for those who had not progressed to CKD stage V; kidney survival time was defined as the time from diagnosis to CKD stage V or the last follow-up; disease activity was assessed by the Pediatric Vasculitis Activity Score (PVAS) (13). Renal complete remission (CR) was defined as negative for proteinuria amount of less than 150 mg/24 h, urine red blood cells were less than 10/HPF, and serum creatinine level was stable; renal partial remission (PR) was defined as a 50% decrease in the daily amount of proteinuria, a decrease in urine red blood cells, and a stable serum creatinine level; nonremission was defined as those that did not meet the above criteria.

Kidney pathologies were classified into focal, crescentic, sclerotic, and mixed subtypes according to Berden’s classification (14). Briefly, samples with ≥50% normal glomeruli were classified as focal; those with ≥50% cellular crescentic glomeruli as crescentic; those with ≥50% sclerotic glomeruli as sclerotic; and those with a combination of normal, crescentic, and sclerotic glomeruli, and all occurring in <50% of glomeruli as mixed.

### Statistical Analysis

Continuous variables with normally distributed measurement were expressed as mean ± standard deviation (SD), evaluated by the Student’s *t*-test. Continuous variables with nonnormal distributed measurement were expressed as median [interquartile range (IQR)], evaluated by the Mann–Whiney *U* test or Wilcoxon rank-sum test. Categorical variables were expressed as percentages and were tested by the Person Chi-square test or Fisher’s exact test. Logistic regression analysis was applied to the multivariate analysis of renal remission, and Cox regression analysis was used to study the risk factors of progressing to ESRD. Kaplan–Meier survival curves and the Log-rank test were applied to compare renal survival among four renal pathological classification and three renal remission-induction responses. All differences were considered statistically significant at *p* < 0.05.

## Results

### Main Characteristics at the Time of Diagnosis of the 48 Patients With MPA and GPA

The baseline data of 48 patients with childhood-onset ANCA-associated vasculitis were summarized in **Table 1**. Most patients were women (81.25%), the mean age at diagnosis was 10.62 ± 3.53 years, and the median time from onset to diagnosis was 1 month. MPA was predominant (91.7%) with just 4 patients with GPA in this cohort; 70.8% of patients showed eGFR <60 ml/min/1.73 m^2^ at diagnosis. Renal involvement was found in 93.75% of patients, as manifested with proteinuria, hematuria, hypertension, and renal insufficiency. The respiratory system was involved in 23% of patients, including 2% with pulmonary nodules and 21% with pulmonary hemorrhage, which was significantly higher in GPA than in MPA (*p* = 0.025). ESR was much faster in MPA than in GPA (*p* = 0.007).

**Table 1:** Main characteristics at the time of diagnosis of the 48 patients.

	MPA (*n* = 44)	GPA (*n* = 4)	*p*-value
**Women (*n* (%))**	34 (77)	3 (75)	0.661
**Age at diagnosis (years)**	11 (0.8–18)	12 (4.8–17)	0.614
**Time from onset to diagnosis (median (months))**	1 (0.1–72)	0.875 (0.25–1)	0.586
**Renal features**			
**Serum creatinine (µmol/L)**	317.29 ± 290.11	145.50 ± 131.51	0.25
**24 h urine protein (mg/24 h)**	1,610.97 ± 1,437.23	2,125.90 ± 3,438.13	0.589
**Nephrotic-range proteinuria (*n* (%))**			
**Hypertension (*n* (%))**	24 (55)	1 (25)	0.338
**eGFR level (*n* (%))**			0.802
eGFR >90 (ml/min/1.73** **m^2^)	9 (20)	2 (50)	
eGFR** **=** **60–90 (ml/min/1.73** **m^2^)	3 (7)	0 (0)	
eGFR** **=** **30–60 (ml/min/1.73** **m^2^)	14 (32)	0 (0)	
eGFR** **=** **15–30 (ml/min/1.73** **m^2^)	11 (25)	1 (25)	
eGFR <15 (ml/min/1.73** **m^2^)	7 (16)	1 (25)	
**Respiratory system (*n* (%))**			
**Pulmonary hemorrhage**	7 (16)	3 (75)	0.025
**Pulmonary nodules**	0 (0)	1 (25)	0.085
**Eye involvement (*n* (%))**	1 (2)	0 (0)	1.000
**ENT involvement (*n* (%))**	0 (0)	1 (25)	0.083
**Neural involvement (*n* (%))**	1 (2)	0 (0)	1.000
**PVAS scores**	12.61 ± 4.45	12.50 ± 6.40	0.962
**C3 (g/L)**	0.91 ± 0.27	0.85 ± 0.20	0.703
**C4 (g/L)**	0.22 ± 0.08	0.18 ± 0.04	0.399
**ESR (mm/h)**	65 ± 41	14 ± 11	0.007
**ANCA (IF) (*n* (%))**			0.039
Negative	7 (16)	1 (25)	
C-ANCA	4 (9)	2 (50)	
P-ANCA	33 (75)	1(25)	
**ANCA (ELISA) (*n* (%))**			0.068
Negative	10 (23)	1(25)	
MPO-ANCA	30 (68)	1 (25)	
PR3-MPO	4 (9)	2 (50)	
**Kidney histopathology (*n* (%)) (Total calculated as the patients with renal biopsy)**			1.000
Focal	3 (10)	0 (0)	
Crescentic	20 (65)	2 (100)	
Sclerotic	6 (19)	0 (0)	
Mixed	2 (6)	0 (0)	
**Treatment (*n* (%))**			0.031
Glucocorticoids	9 (21)	2 (50)	
Glucocorticoids+CYC	20 (45)	0 (0)	
Glucocorticoids+CYC+PE	10 (23)	0 (0)	
Glucocorticoids+CYC+PE+RTX	5 (11)	2 (50)	

PVAS, Pediatric Vasculitis Activity Score; IF, immunofluorescence; CYC, cyclophosphamides; PE, plasma exchange; RTX, rituximab.

### Main Characteristics at the Time of Diagnosis of the 33 Patients Based on Kidney Pathology

The crescentic subtype was the most frequent (66.7%) form among 33 patients who received kidney biopsies. As shown in **Table 2**, the main characteristics were not significantly different at the time of diagnosis among the four pathological subtypes.

**Table 2:** Clinical features at the time of diagnosis of patients with different histopathological subtypes.

	Focal (*N* = 3)	Crescentic (*N* = 22)	Sclerotic (*N* = 6)	Mixed (*N* = 2)	*p*-value
**Women (*n* (%))**	2 (67)	16 (73)	6 (100)	0 (0)	0.055
**Age at onset (years)**	11.67 ±1.15	9.99 ±3.31	10.78 ±1.65	10.15 ±4.03	0.799
**Time from onset to biopsy**	0.5 (0.1–6)	1 (0.1–6)	0.5 (0.1–3)	36.1 (0.25–72)	0.701
**Clinical type**					1.000
MPA (*n* (%))	3 (100)	20 (91)	6 (100)	2	
GPA (*n* (%))	0 (0)	2 (9)	0 (0)	0	
**ANCA (IF) (*n* (%))**					0.128
Negative	1 (33)	4 (18)	0 (0)	2	
C-ANCA	0 (0)	3 (14)	0 (0)	0	
P-ANCA	2 (67)	15 (68)	6 (100)	0	
**ANCA (ELISA) (*n* (%))**					0.162
Negative	1 (33)	6 (27)	0 (0)	2	
MPO	2 (67)	13 (59)	6 (100)	0	
PR3	0 (0)	3 (14)	0 (0)	0	
**eGFR level (*n* (%))**					0.022
eGFR >90	2 (67)	3 (14)	0 (0)	1 (50)	
eGFR** **=** **60–90	1 (33)	1 (5)	1 (17)	0 (0)	
eGFR** **=30–60	0 (0)	6 (27)	3 (50)	1 (50)	
eGFR** **=** **15–30	0 (0)	10 (45)	0 (0)	0 (0)	
eGFR <15	0 (0)	2 (9)	2 (33)	0 (0)	
**Serum creatinine (µmol/L)**	55 ± 32.5	327.5 ± 266.6	396.2 ± 389.5	132 ± 152.7	0.406
**24h urine protein (mg/24h)**	1,040 ± 650.5	1,642.3 ± 1,870.7	2,502.1 ± 1,158.9	3,384.9 ± 3,014	0.412
**ESR (mm/H)**	74.5 ± 41.7	67.6 ± 49.8	75.5 ± 41.6	68 ± 69.3	0.986
**C3 (g/L)**	0.93 ± 0.46	0.93 ± 0.28	0.95 ± 0.18	0.57 ± 0.13	0.395
**C4 (g/L)**	0.23 ± 0.11	0.21 ± 0.76	0.33 ± 0.14	0.11 ± 0.02	0.068
**PVAS**	11 ± 1.4	13.0 ± 3.9	13.5 ± 3.2	13 ± 4.2	0.876
**Treatment (*n* (%))**					0.217
Glucocorticoids	1 (33)	1 (5)	0 (0)	1 (50)	
Glucocorticoids+CTX	2 (67)	12 (55)	2 (33)	0 (0)	
Glucocorticoids+CTX+PE	0 (0)	4 (18)	2 (33)	1 (50)	
Glucocorticoids+CTX+PE+RTX	0 (0)	5 (23)	2 (33)	0 (0)	

PVAS, Pediatric Vasculitis Activity Score; IF, immunofluorescence; CYC, cyclophosphamides; PE, plasma exchange; RTX, rituximab.

### Different Responses to Induction Treatment and Predictive Factors Analysis of Baseline Data

After up to 6-month induction treatment, complete remission was achieved in 8 patients (16.7%), partial remission in 19 patients (39.6%), and nonremission in 21 patients (43.7%). The baseline data were compared among the three groups; significant differences were found in levels of serum creatinine and C3, hypertension, eGFR grades, and PVAS score (**Table 3**). eGFR <60 ml/min/1.73 m^2^ (*p* = 0.001) and hypertension (*p* = 0.002) were independent predictors for no response to induction treatment by multivariate analysis (**Table 4**).

**Table 3:** The baseline data at diagnosis and treatments in patients with different renal responses to induction treatment.

	Complete remission (*N* = 8)	Partial remission (*N* = 19)	Nonremission (*N* = 21)	*p*-value
**Women (*n* (%))**	7 (87.5)	14 (73.7)	16 (76.2)	0.816
**Age at diagnosis (years)**	12 (2.3–17)	11 (2.5–18)	11 (8–16)	0.222
**Time from onset to diagnosis (median (months))**	0.875 (0.1–2)	1 (0.25–72)	1 (0.1–36)	0.592
**Renal features**				
Serum creatinine (µmol/L)	102.20 ± 147.59	184.87 ± 221.49	486.32 ± 269.27	<0.001
24** **h urine protein (mg/24** **h)	717.113 ± 990.41	1,579.61 ± 1,584.66	2,077.94 ± 1,764.77	0.129
Nephrotic-range proteinuria (*n* (%))	0 (0)	3 (16.7)	6 (27.3)	0.263
Hypertension (*n* (%))	0 (0)	9 (50)	15 (68.2)	0.003
**eGFR level (*n* (%))**				<0.001
eGFR >90 (ml/min/1.73** **m^2^)	6 (75)	5 (26.3)	0 (0)	
eGFR** **=** **60–90 (ml/min/1.73** **m^2^)	1 (12.5)	2 (10.5)	0 (0)	
eGFR** **=** **30–60 (ml/min/1.73** **m^2^)	0 (0)	9 (47.4)	5(23.8)	
eGFR** **=** **15–30 (ml/min/1.73** **m^2^)	1 (12.5)	3 (15.8)	8 (38.1)	
eGFR <15 (ml/min/1.73** **m^2^)	0 (0)	0 (0)	8 (38.1)	
**C3 (g/L)**	1.04 ± 0.32	0.97 ± 0.21	0.77 ± 0.24	0.027
**C4 (g/L)**	0.21 ± 0.06	0.19 ± 0.07	0.23 ± 0.10	0.54
**ESR (mm/h)**	35 ± 46.88	62.69 ± 38.63	67.25 ± 40.87	0.22
**PVAS scores**	9 (1–15)	12 (3–20)	12 (10–27)	0.002
**ANCA (IF) (*n* (%))**				0.379
Negative	2 (25)	2 (11.1)	4 (18.2)	
C-ANCA	2 (25)	3 (16.7)	1 (4.5)	
P-ANCA	4 (50)	14 (72.2)	16 (77.3)	
**ANCA (ELISA) (*n* (%))**				0.288
Negative	3 (37.5)	3 (16.7)	5 (22.7)	
MPO-ANCA	3 (37.5)	13 (66.7)	15 (72.7)	
PR3-MPO	2 (25)	3 (16.7)	1 (4.5)	
**Kidney histopathology (*n* (%))**	(Total calculated as the patients with renal biopsy)			0.156
Focal	2 (66.7)	2 (12.5)	0 (0)	
Crescentic	1 (33.3)	11 (68.75)	9 (64.29)	
Sclerotic	0 (0)	2 (12.5)	4 (28.57)	
Mixed	0 (0)	1 (6.25)	1 (7.14)	
**Treatment (*n* (%))**				0.183
Glucocorticoids	4 (50)	3 (15.8)	4 (19.0)	
Glucocorticoids+CYC	4 (50)	10 (52.6)	6 (28.6)	
Glucocorticoids+CYC+PE	0 (0)	3 (15.8)	7 (33.3)	
Glucocorticoids+CYC+PE+RTX	0 (0)	3 (15.8)	4 (19.0)	

PVAS, Pediatric Vasculitis Activity Score; IF, immunofluorescence; CYC, cyclophosphamides; PE, plasma exchange; RTX, rituximab.

**Table 4:** Multivariate analysis of risk factors associated with nonremission in 48 patients after remission-induction therapies.

Factors	*p*-value	OR value	95% CI
**Women**	0.544	1.737	0.292–10.325
**Hypertension**	0.002	19.574	3.036–126.219
**Nephrotic-range proteinuria**	0.658	1.598	0.200–12.751
**eGFR <60 ml/min/1.73 m^2^**	0.001	28.020	3.786–207.364
**PVAS scores**	0.562	0.544	0.069–4.261
**Age >11**	0.833	0.847	0.181–3.958

PVAS, Pediatric Vasculitis Activity Score.

### Renal Outcomes and Risk Factors Associated With Progression to ESRD

The median time of follow-up was 24.5 months (0–228 months). A total of 24 patients (50%) progressed to ESRD. The mean time they progressed to ESRD was (13.04 ± 15.83) months, and the median time was 7.5 months (0–58 months). A total of 8 patients reached ESRD at the time of diagnosis, and 4 patients progressed to ESRD in the first 6 months after diagnosis. The other 12 patients progressed to ESRD thereafter; among them, 4 patients showed partial remission postinduction treatment, and 8 patients showed nonremission.

The baseline data of 48 patients were applied to Cox regression analysis to identify the risk factors of ESRD. As shown in **Table 5**, hypertension, eGFR <60 ml/min/1.73 m^2^, PVAS >11, and nephrotic proteinuria at diagnosis were significantly associated with ESRD in univariate analysis, but only hypertension (*p* = 0.001) and eGFR <60 ml/min/1.73 m^2^ (*p* = 0.013) were proved to be independent risk predictors of ESRD in multivariate Cox regression analysis.

**Table 5:** Analysis of risk predictors at diagnosis associated with progression to ESRD in follow-up.

Factors	Univariate analysis			Multivariate analysis		
	*p*-value	OR	95% CI	*p*-value	OR	95% CI
**Women**	0.767	0.869	0.344–2.196	0.879	1.094	0.344–3.484
**Age >11**	0.367	0.690	0.309–1.544	0.568	1.344	0.487–3.711
**Hypertension**	<0.001	9.675	3.208–29.18	0.001	13.12	2.995–57.45
**Nephrotic proteinuria**	0.028	2.596	1.107–6.086	1.000	1.000	0.350–2.856
**eGFR <60 ml/min/1.73 m^2^**	0.012	6.374	1.493–27.21	0.013	9.597	1.625–56.67
**PVAS score >11**	0.004	8.593	1.999–36.93	0.598	1.640	0.261–10.33
**PE**	0.772	0.884	0.383–2.040	0.910	0.974	0.373–2.409

PVAS, Pediatric Vasculitis Activity Score; PE, plasma exchange; RTX, rituximab.

**Figure 1:**
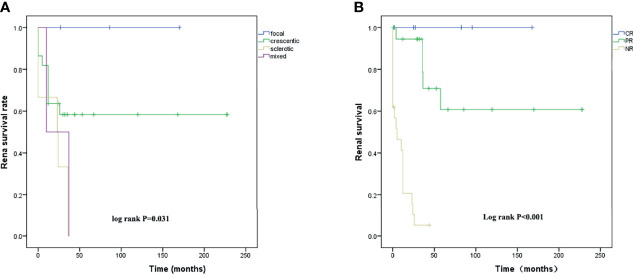
**(A)** Renal survival analysis K-M curves of the time from diagnosis to ESRD or last follow-up in patients with different renal pathological subtypes and **(B)** in patients with different induction remission responses after 6-month treatment.

Renal biopsies were performed in 33 patients, including 9 patients of crescentic subtype, 6 of sclerotic subtype, and 2 of mixed subtype. The renal survival curves of different pathological subtypes are shown in **Figure 1A**; a significant difference was found among four renal pathological subtypes (*p* = 0.031). The sclerotic subtype showed the worst and the focal showed the best outcomes. The renal outcomes at final evaluation in patients with different pathological subtypes are shown in **Supplementary Figure S1**.

All patients of focal subtype remained under normal renal function, but all patients of sclerotic and mixed subtypes progressed to ESRD. Since the patients of the crescentic subtype showed variable outcomes, we compared their baseline data between those who remained under normal renal function (eGFR >90 ml/min/1.73 m^2^) and those who progressed to ESRD (eGFR <15 ml/min/1.73 m^2^) and found significant differences in age, serum creatinine level, eGFR level, and response to remission-induction treatment (**Table 6**).

**Table 6:** The clinical data at diagnosis and response to induction treatment in patients of a crescentic subtype with different renal outcomes at the endpoint.

	eGFR >90 ml/min/1.73 m^2^	eGFR <15 ml/min/1.73 m^2^	*p*-value
**Women (*n*, %)**	6 (75)	7 (78)	1.000
**Age >11**	12±2.8	8.6±2.7	0.022
**Serum creatinine (µmol/L)**	159.2±140.4	552.2±257.1	0.002
**24 h urine protein (mg/24 h)**	1,370.2±1,706	2,332.7;±2,335.1	0.353
**PVAS score**	11.5±4.8	13.1 ± 4.2	0.159
**Hypertension (*n*, %)**	4 (50%)	5 (56)	0.614
**eGFR level (*n*, %)**			0.001
eGFR >90 (ml/min/1.73** **m^2^)	3 (37.5%)	0 (0)	
eGFR** **=** **60–90 (ml/min/1.73** **m^2^)	1 (12.5)	0 (0)	
eGFR** **=** **30–60 (ml/min/1.73** **m^2^)	3 (37.5)	0 (0)	
eGFR** **=** **15–30 (ml/min/1.73** **m^2^)	1 (12.5)	7 (78)	
eGFR <15 (ml/min/1.73** **m^2^)	0 (0)	2 (22)	
**Treatment (*n*, %)**			0.424
Glucocorticoids+CTX	7 (87.5)	5 (55.6)	
Glucocorticoids+CTX+PE	1 (12.5)	3 (33.3)	
Glucocorticoids+CTX+RTX	0 (0)	1 (11.1)	
**Remission at 6-month (*n*, %)**			<0.001
Complete remission	2 (25)	0 (0)	
Partial remission	6 (75)	1 (11.1)	
Nonremission	0 (0)	8 (88.9)	

PVAS, Pediatric Vasculitis Activity Score; CYC, cyclophosphamides; PE, plasma exchange; RTX, rituximab.

To evaluate the effect of different responses to induction treatment on renal survival, Kaplan–Meier survival curves were used to assess the time to ESRD in 48 patients. Those patients with complete remission showed the best renal outcome and those with nonremission showed the worst (**Figure 1B**).

About 7 patients were treated with rituximab (RTX); 3 cases with crescentic subtype obtained improvement of eGFR from 19 to 47 ml/min/1.73 m^2^, 27 to 79 ml/min/1.73 m^2^, and 59 to 67 ml/min/1.73 m^2^, respectively. Two cases with crescentic subtype and 2 cases with sclerotic subtype still showed eGFR deterioration (**Supplementary Figure S2**).

## Discussion

AAV is a severe autoimmune disorder mainly affecting the kidney, lung, nose, paranasal sinus, skin, etc. (15). It mainly occurs in adults and rarely in children. So far, a few large cohorts are focusing on the long-term outcome of pediatric patients with AAV. Our study summarized the clinicopathological features, their relationship with response to induction treatment, and renal long-term outcome of pediatric AAV in a single center. The results showed that MPA accounted for the overwhelming majority (91.7%) of AAV in Chinese children, and female patients also made up the majority (81.25%) of AAV. The demographic and AAV-type distribution characteristics were consistent with other previous studies on Chinese and other East Asian children (10, 11, 16, 17). These were different from adult patients, suggesting a somewhat male predominance (18). It is interesting to note that an MPA majority not only existed in studies comprising Chinese pediatric (97.1%) (10) and adult patients (83.64%) (19) but also in Korean (59.4%) (20) and Japanese patients with AAV (21). It was different from AAV in Europe and North America where a GPA majority was more common (21). Renal involvement is much higher than pulmonary involvement in MPA patients. Similarly, our study showed renal and pulmonary involvement in 93.75% and 23% of patients, respectively.

As shown in the present paper, the effect of induction therapy significantly affected renal survival in patients with AAV. Currently, glucocorticoids combined with cyclophosphamide remain the “gold standard” treatment for induction therapy. Mycophenolate mofetil and azathioprine are the main immunosuppressants for maintenance therapy. In recent years, rituximab, immunoadsorption, and plasma exchange have also been successfully used in the treatment of AAV (3). Other biologics like anti-TNF-α and anti-BLyS monoclonal antibodies have been tried in the treatment of AAV. The overall remission rate was 56.3% after 6-month induction treatment in our patients, which was significantly lower than that in previously reported AAV cohorts (9, 10, 22). As shown in our study, baseline eGFR <60 ml/min/1.73 m^2^ was an independent predictor of nonremission and found in a percentage as high as 71% of patients. Therefore, the reason for the lower remission rate of induction treatment was probably due to the higher proportion of eGFR <60 ml/min/1.73 m^2^ at diagnosis.

Around 50% of patients progressed to ESRD, higher than in other reports showing only between 20% and 45% (4–10). Through multivariate Cox regression analysis, we identified eGFR <60 ml/min/1.73 m^2^ and hypertension at the time of diagnosis as independent risk predictors for progression to ESRD. Our results confirmed the poor prognostic value of decreased eGFR at diagnosis, which was consistent with previous studies (4, 8–10, 23). Additionally, this study revealed a new finding that hypertension at diagnosis was also an independent risk predictor for progression to ESRD in childhood-onset AAV. Moreover, this study also identified nephrotic-range proteinuria and PVAS >11 at the time of diagnosis as risk factors for ESRD in univariate analysis. PVAS was established in 2012 when redefining the Birmingham Vasculitis Activity Score (BVAS) components and adding eight pediatric items (13). The present study also confirmed high PVAS as a risk factor of renal long-term outcome in pediatric AAV patients (24–26). A total of eight patients reached ESRD at the time of diagnosis, and four patients progressed to ESRD in the periods of remission-induction treatment. Considering that immunosuppressants were not necessarily needed in these cases, they received a shorter period of induction therapy to avoid adverse effects, and this might have an impact on renal outcomes.

Our results also demonstrated that renal pathological patterns were significantly associated with long-term renal outcomes, as shown in adult AAV patients. There were studies even showing that renal pathological patterns affected the outcome more than baseline eGFR (27, 28). As in adults, those with a sclerotic subtype rapidly progressed to ESRD. Sclerotic lesions were thought to result not only from vasculitis but also from other factors, such as aging, atherosclerosis, dyslipidemia, and hypertension (9, 29). Patients with focal subtype showed a favorable renal outcome. Interestingly, the outcome of those with crescentic subtype turned out to see many variations, and whether they restored to normal eGFR levels or progressed to ESRD (CKD stage V) was significantly associated with baseline eGFR levels and with response to induction treatment. There were three patients with eGFR levels between 30 and 60 ml/min/1.73 m^2^ and one patient with an eGFR level between 15 and 30 ml/min/1.73 m^2^; they all finally returned to normal eGFR levels following a 6-month remission-induction treatment. These results demonstrated the importance of treatment responsiveness in affecting the outcome. Of course, the active lesions were more likely to be reversible if treated in a timely and effective manner (30). In addition to glucocorticoid and CYC, RTX was also proven to be as effective as CYC in remission-induction and relapse treatment of AAV (31, 32). Among seven serious AAV patients treated with RTX, three patients obtained a significant improvement in eGFR and proteinuria, which demonstrated the promising prospects of RTX in the treatment of pediatric patients with AAV.

The advantages of this study were based on the fact that it represented the largest pediatric cohort of AAV in Chinese children and provided actual clinicopathological features different from adults and children in the EU and North America. Secondly, our study demonstrated that eGFR <60 ml/min/1.73 m^2^ and hypertension at diagnosis were independent predictors for progression to ESRD. Moreover, the study particularly pointed out the importance of induction treatment responsiveness in affecting the outcome of AAV. Through multivariate logistic regression analysis, hypertension and eGFR <60 ml/min/1.73 m^2^ at diagnosis were also considered risk factors associated with failure of induction therapies. Thus, these risk factors might affect the long-term renal outcomes by influencing the response to induction treatment. However, the limitations of this study lie mainly in its retrospective nature, and the total number of included cases is not large enough to allow further statistical analysis. In addition, since we included only hospitalized patients, a selection bias may exist in that some patients with mild AAV in the outpatient were omitted.

In conclusion, our study demonstrates that women, MPA, and crescentic subtypes are predominant in pediatric AAV in China. Initial renal failure (eGFR <60 ml/min/1.73 m^2^), hypertension, sclerotic pathological pattern at diagnosis, and nonremission to induction treatment are predictors of poor long-term renal outcome. Apparently, AAV remains a rough challenge to the pediatric community.

## Data Availability Statement

The original contributions presented in the study are included in the article/**Supplementary Material**, further inquiries can be directed to the corresponding author/s.

## Ethics Statement

The studies involving human participants were reviewed and approved by the Human Ethics Committee of Tongji Hospital, Tongji Medical College, Huazhong University of Science and Technology. Written informed consent to participate in this study was provided by the participants’ legal guardian/next of kin.

## Author Contributions

All authors contributed to the intellectual content of this manuscript and approved the final manuscript as submitted. JY collected data, performed the statistical analysis, and drafted the manuscript with the help of YX, YY, LQZ, LWZ, XY, FY, YH, JP, YL, YXC, and JZ. HY, LQ, YZ, YC, TL, and JT were responsible for the renal pathological interpretation and patients’ care. JZ interpreted the data and revised the article for important intellectual content. All authors have critically read and approved the manuscript.

## Funding

This work was supported by a grant (No. 81873596) from the National Natural Science Foundation of China to JZ.

## Conflict of Interest

The authors declare that the research was conducted in the absence of any commercial or financial relationships that could be construed as a potential conflict of interest.

## Publisher’s Note

All claims expressed in this article are solely those of the authors and do not necessarily represent those of their affiliated organizations, or those of the publisher, the editors and the reviewers. Any product that may be evaluated in this article, or claim that may be made by its manufacturer, is not guaranteed or endorsed by the publisher.
