# Supplementary material for: Clinical and Renal Histology Findings and Different Responses to Induction Treatment Affecting the Long-Term Renal Outcomes of Children With ANCA-Associated Vasculitis: a Single-Center Cohort Analysis
Source: Front Immunol. 2022 Apr 14;13:857813. doi: 10.3389/fimmu.2022.857813 (PMC9047757; doi:10.3389/fimmu.2022.857813)
Supplement: Supplementary file 1 [file Image_1.pdf]

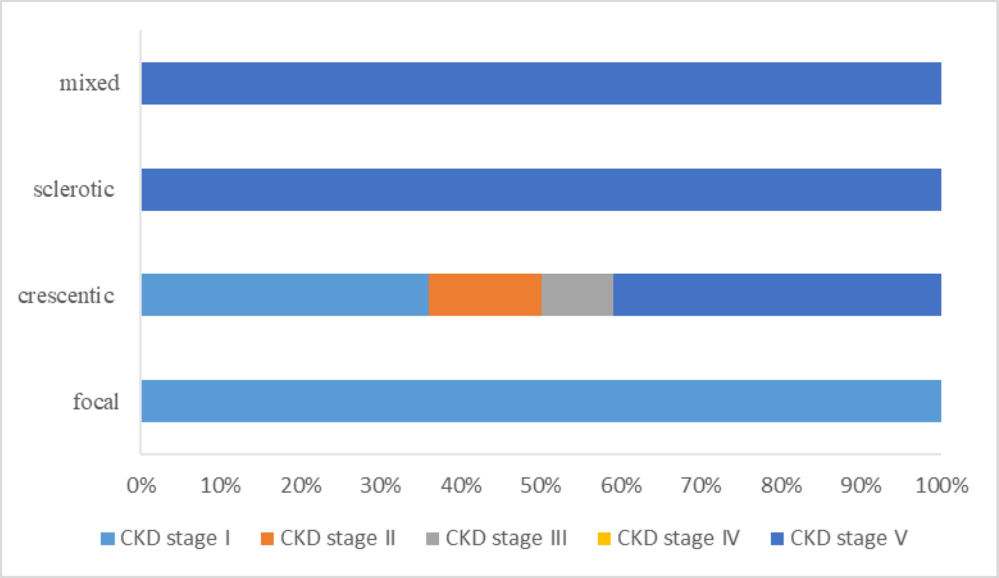

Supplementary Figure 1 The renal outcomes at final evaluation in patients with different pathological subtypes at diagnosis
