# Supplementary figures and images for: Clinical and Renal Histology Findings and Different Responses to Induction Treatment Affecting the Long-Term Renal Outcomes of Children With ANCA-Associated Vasculitis: a Single-Center Cohort Analysis
Source: Front Immunol. 2022 Apr 14;13:857813. doi: 10.3389/fimmu.2022.857813 (PMC9047757; doi:10.3389/fimmu.2022.857813)

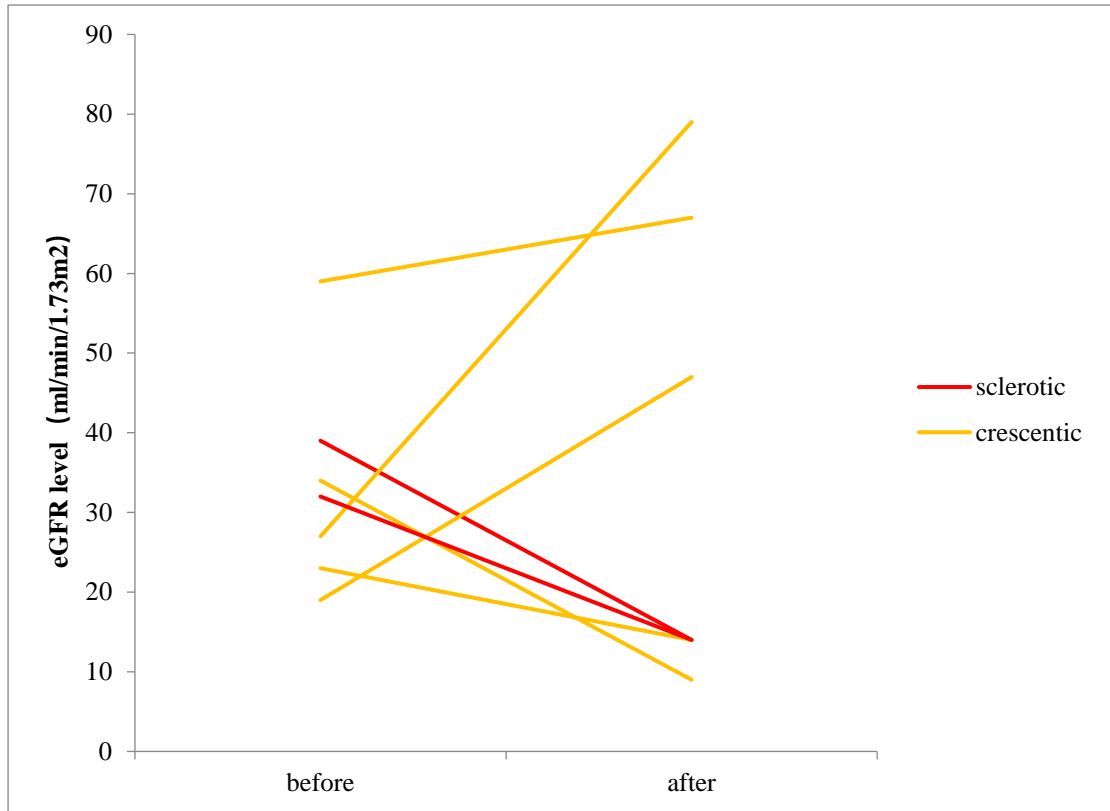

**Supplementary Figure 2** The eGFR level of the patients before and after RTX treatment

Supplement: Supplementary file 2 [file Image_2.pdf]
